# Application of Machine Learning Based on Structured Medical Data in Gastroenterology

**DOI:** 10.3390/biomimetics8070512

**Published:** 2023-10-28

**Authors:** Hye-Jin Kim, Eun-Jeong Gong, Chang-Seok Bang

**Affiliations:** 1 Department of Internal Medicine, College of Medicine, Hallym University, Chuncheon 24253, Republic of Korea; khyejin1027@hanmail.net (H.-J.K.); gong-eun@hanmail.net (E.-J.G.); 2 Institute for Liver and Digestive Diseases, Hallym University, Chuncheon 24253, Republic of Korea; 3 Institute of New Frontier Research, College of Medicine, Hallym University, Chuncheon 24253, Republic of Korea

**Keywords:** machine learning, artificial intelligence, gastroenterology

## Abstract

The era of big data has led to the necessity of artificial intelligence models to effectively handle the vast amount of clinical data available. These data have become indispensable resources for machine learning. Among the artificial intelligence models, deep learning has gained prominence and is widely used for analyzing unstructured data. Despite the recent advancement in deep learning, traditional machine learning models still hold significant potential for enhancing healthcare efficiency, especially for structured data. In the field of medicine, machine learning models have been applied to predict diagnoses and prognoses for various diseases. However, the adoption of machine learning models in gastroenterology has been relatively limited compared to traditional statistical models or deep learning approaches. This narrative review provides an overview of the current status of machine learning adoption in gastroenterology and discusses future directions. Additionally, it briefly summarizes recent advances in large language models.

## 1. Introduction

Artificial intelligence (AI) is a technology that is rapidly being adopted in many industries, primarily to improve diagnostic performance, precision, analysis time, and efficiency, as well as to reduce costs [1]. The term AI was coined by John McCarthy at a lecture at Dartmouth College in 1956 [2]. With the improvement of computers and contributions from other disciplines, the field of AI has advanced remarkably, recently emerging as its own field [3]. Big data has facilitated the utilization of AI, especially machine learning (ML), which is a subcategory of AI [4]. Among AI models, deep learning models are on the rise. These models have been adopted for the analysis of unstructured data (image, documents, videos, or audio files., etc.). However, besides the recent advancement of deep learning models, traditional machine learning (ML) models have the potential to improve healthcare efficiency in a variety of ways, especially for structured data [5,6].

ML is a discipline in computer science wherein computers are programmed to learn patterns from data. The learning itself is based on a set of mathematical rules and statistical assumptions [7]. ML methods are known to make more accurate and reliable predictions than traditional statistical methods by making non-linear predictions from big data. [8]. To predict a closing situation, ML algorithms identify patterns in data and associate those patterns with distinct classes of records. Therefore, ML methods are expected to be useful in estimating clinical diagnoses or predicting patient outcomes based on large amounts of clinical data [9].

Recently, ML methods have been widely used in medicine. In the field of gastroenterology, physicians demand the ability to analyze diverse types of data and incorporate them into predictions for disease risk, diagnosis, prognosis, and appropriate treatments [5,6]. From the perspective of precision medicine, the ML approach is expected to be used as a tool for clinical benefit and treatment success by predicting individualized diagnoses and clinical courses, as it is more accurate and precise than traditional statistical analysis [10]. It has been predicted that the usage of ML methods will increase in clinical medicine due to current expectations and technological advancements in AI, particularly deep learning. However, there are few reported uses of ML methods in the field of gastroenterology. The aim of this study was to (1) introduce general aspects of ML analysis methodologies, (2) describe an ML application in the field of gastroenterology, and (3) discuss the challenges for clinical application and future perspectives of ML for clinicians unfamiliar with ML methods. Additionally, recent advances in large language models will be concisely summarized.

## 2. ML Technology

**Figure 1:**
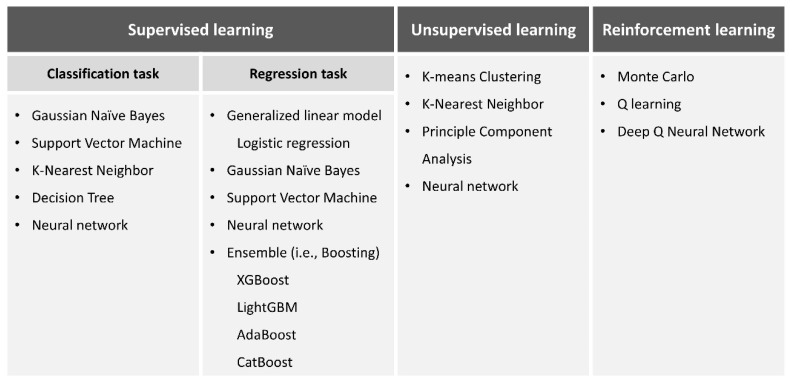
Schematic view of representative machine learning models.

**Figure 2:**
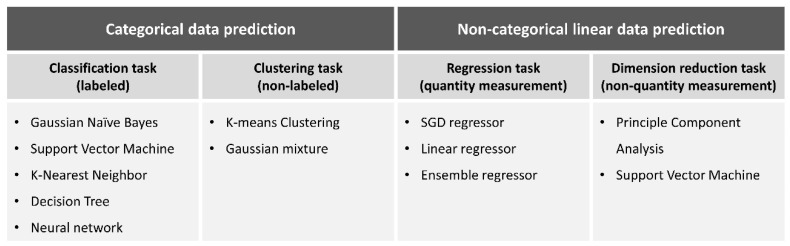
Basic discipline for selecting machine learning models in each task.

ML is a mathematical AI algorithm capable of predicting precise outcomes in uncertain situations without explicit programming. Examples of ML include Bayesian inferences, decision trees, support vector machines (SVM), deep neural networks, and ensemble methods (bagging or boosting). In essence, ML is an applied statistical technique known for its high accuracy. Figure 1 describes representative ML models, while Figure 2 illustrates the fundamental principles for selecting ML models for various tasks.

**Figure 3:**
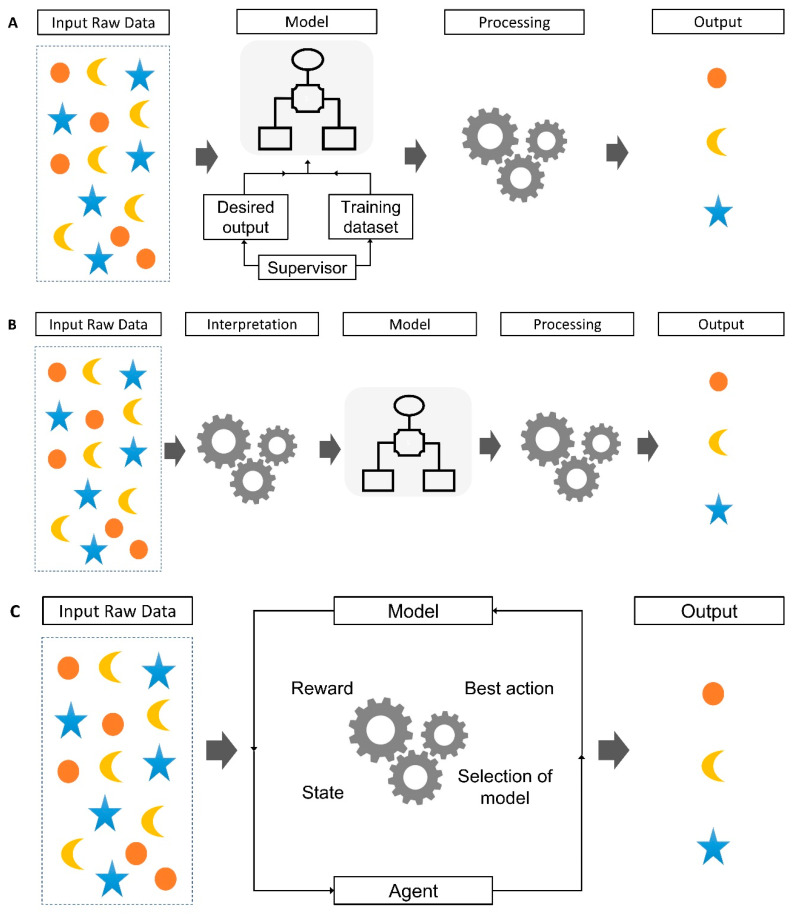
Three main categories of learning tasks in machine learning. (**A**) Supervised learning, (**B**) unsupervised learning, (**C**) reinforcement learning.

ML methods possess the capability to automatically analyze data, recognize patterns autonomously, and, after a learning phase, apply these learned patterns even when presented with new data [11]. Based on the nature of the available data, there are three primary categories of learning tasks: supervised learning (A), unsupervised learning (B), reinforcement learning (C), as shown in Figure 3. 

### 2.1. Supervised Learning

Supervised learning begins with the objective of prediction, which involves known output or target values [12]. The most common supervised tasks are “classification” which categorizes data, and “regression” which fits data [13]. Supervised learning, encompassing regression and classification, is an analytical approach that identifies patterns or features by learning from the labeled output data (where ground truth exists) and applies this knowledge to new input data to make accurate predictions. Classification tasks encompass SVM, discriminant analysis, naïve Bayes, nearest neighbor, and neural networks, while regression tasks include GLM, linear regression, ensemble method, decision trees, and neural networks [14].

### 2.2. Unsupervised Learning

In unsupervised learning, the model is provided with only the input data during training. In other words, we are asking the model to learn without a predicted correct answer.

Feature learning allows us to discover unknown patterns through data analysis. As a result, we become capable of classifying new data when presented as input. For instance, we can learn to categorize data based on similarities, patterns, differences, and other factors among the data points. Typical unsupervised learning methods encompass clustering, density, estimation, dimensionality reduction, anomaly detection, and more [13].

### 2.3. Reinforcement Learning

Reinforcement learning draws its foundation from behavioral psychology. It is a type of ML that provides machines with the capability to autonomously evaluate the best course of action in a given situation or environment, aiming to enhance the efficiency of computer programs. Representative examples of reinforcement learning include Monte Carlo, Q learning, and deep Q neural networks [15].

### 2.4. Recent Advancement of ML Analysis Models

The fundamental principle of problem solving involves making optimal predictions based on input data through mathematical calculations. 

Overfitting is a modeling error that occurs when a learning model becomes overly tailored to the training data, resulting in predictions that do not generalize well to new data. This happens because the model learns not only the underlying patterns, but also the noise present in the data. Such a model is considered overparameterized and often overly complex relative to the size of the training dataset. Naturally, it fits the training data perfectly [16]. 

There are two approaches to dealing with overfitting. The first is to modify the model, such as by employing a less complex backbone model, reducing parameters, or increasing learning restrictions. The second step is to manipulate the data, such as by eliminating unnecessary features, increasing the amount of data to improve the model, or reducing noise in the data through preprocessing [16].

Conversely, underfitting occurs when the model is too simplistic to capture the underlying features of the data. To address this issue, we opt for a more powerful model with a greater number of parameters. We may also relax the model’s training constraints and augment the richness of the dataset [16].

ML results can be biased and inaccurate if data distribution is highly imbalanced or if underfitting and overfitting occur. To mitigate this issue, ensemble learning methods can be employed [17]. 

Ensemble methods are one of the representative techniques in supervised learning, which involve combining predictions from multiple models to generate a final result. By aggregating different classifiers into a large meta-classifier, the objective is to achieve better generalization performance than individual classifiers [18]. These techniques include voting for classification and averaging for regression. By partitioning large amounts of a dataset into smaller subsets, training each model independently, and merging the results, you enhance accuracy while reducing errors [19].

Many ML models, including logistic regression, SVM, decision trees, kNN classifiers, and more, have been used to evaluate big data. However, recent advances in ML analytical models continuously improve by employing ensemble techniques to enhance accuracy and predictive power. 

**Figure 4:**
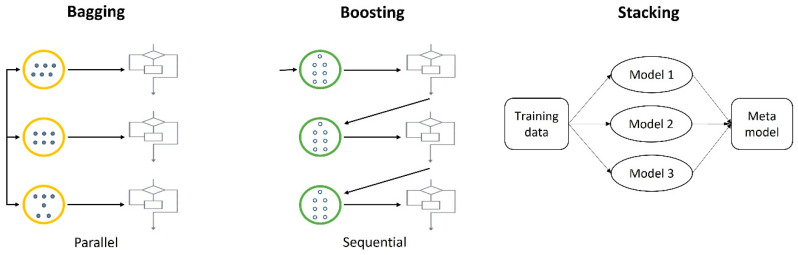
Schematic view of the training technique of ensemble machine learning models.

Ensemble techniques, a category of ML techniques, combine multiple weak learners to create a single strong learner. These ensemble techniques include bagging, which uses a voting method; boosting, which employs a weighted voting method; and stacking, which utilizes predicted values obtained from a single model as training data (Figure 4).

### 2.5. Boosting (Hypothesis Boosting)

Boosting is a ML ensemble technique that combines multiple sequential weak learners to improve prediction or classification performance. This process involves classifying the initially sampled dataset and then resampling it with weighted consideration for the misclassified data [20]. In essence, boosting transforms a weak classifier into a strong one by utilizing these weights. Given its primary use in reducing bias, models with low variance and high bias are suitable candidates for this technique, with gradient boosting and extreme gradient boosting (XGBoost) serving as representative examples.

### 2.6. Bagging

Bagging, which is short for “bootstrap aggregation”, was introduced by Breiman as a technique for reducing the prediction error of learning algorithms [21]. It involves generating multiple versions of a predictor through bootstrap sampling and then using these versions to create an improved predictor. In the case of predicting numeric outcomes, it averages the predictions, while for predicting classes, it employs a multi-voting approach. 

It is a parallel ensemble model because each model is constructed in parallel and independently. It is often used to reduce variance and is therefore suitable for high-variance, low-bias models (such as overfitting models). Random forests are a representative example of this approach.

### 2.7. Stacking (Stacked Generalization)

Stacked generalization is an ensemble method that enables researchers to combine several different prediction algorithms into one [22]. Stacking is meta-trained with the training results of multiple models, allowing it to perform well on training data [23]. However, when data from a different domain or with a different variance than the training data are introduced, the prediction/classification performance of the model significantly decreases. This occurs because stacking involves two stages: 1. generation of models and 2. meta-learning with models’ results, leading to overfitting of the training data [24]. To address this issue, a common approach is to add a second stage of cross-validation, known as cross-validation set-based stacking.

## 3. Application of ML in Gastroenterology

ML models have been applied to predict treatment outcomes or prognoses in certain conditions, analyzing structured data such as electronic medical record formats or tabular data formats. These models have shown promising results in disease diagnosis and prognosis prediction [25]. In most studies, ensemble learning techniques were applied in ML. 

After reviewing approximately 300 papers retrieved using the keywords ‘gastroenterology’ and ‘machine learning’ in PubMed from 2018 to January 2022, we identified 25 papers that aligned with the purpose of our research. Among these, 12 papers utilized ML models for diagnosing and predicting the prognosis of gastric cancer. ML models that incorporated ensemble techniques like XGBoost, gradient-boosting decision tree (GBDT) and gradient-boosting machines (GBM) demonstrated outstanding performance. Accuracy ranged from 0.90 to 0.96 and the area under the curve (AUC) ranged from 0.75 to 0.90. 

### 3.1. General Subjects

Klang E et al. employed 31 clinical and biological characteristics in a study involving 4497 individuals to identify specific risk factors in the emergency department associated with complex acute diverticulitis. They utilized the XGBoost model, which demonstrated an internal test sensitivity of 88% and a negative predictive value of 99% [26]. Yoshii S. et al. developed an ML algorithm based on Lasso and elastic-net regularization techniques. This algorithm aimed to predict *Helicobacter pylori* infection status in endoscopic images using the Kyoto classification of gastritis. With the Kyoto classification system, the overall diagnostic accuracy reached 82.9% [27]. Konishi T et al. utilized 45 clinical factors in a study involving 25,886 patients to construct ML models for assessing the probability of postoperative mortality in gastroduodenal ulcer perforation. The results indicated that Lasso and XGBoost-based ML models outperformed the standard American Society of Anesthesiology score, achieving an AUC of 0.84 for Lasso and 0.88 for XGBoost in the internal test group [28]. Liu Y et al. developed ML-based models to predict the risk of upper gastrointestinal lesions in order to identify patients at high risk for endoscopy. In this study involving 620 patients, they incorporated 48 clinical symptoms, serological findings, and pathological factors. The SVM-based model emerged as the top performer, boasting an accuracy of 93.4% in the training set and 91.2% in the internal test set [29]. Table 1 provides a detailed description of each study.

### 3.2. Gastrointestinal Hemorrhage

In the context of gastrointestinal hemorrhage, Shung DL et al. employed an XGBoost model to predict the risk of intervention or death in 2357 patients with upper gastrointestinal bleeding, utilizing 24 clinical and biological variables. This model achieved an AUC of 0.91 for internal tests and an AUC of 0.90 for external tests. [30]. Herrin J et al. developed ML models to predict antithrombotic-related gastrointestinal hemorrhage. The study included 306,463 patients and 32 clinical factors. The AUC for the prediction of antithrombotic-related gastrointestinal hemorrhage at 6 months was 0.67 for the regularized Cox proportional hazards regression model and the XGBoost model [31]. ML models that predict adverse outcomes in patients with initially stable non-variceal upper gastrointestinal bleeding were created by Seo DW et al. They used 38 clinicopathological characteristics from 1439 patients as input data. The Glasgow-Blatchford and Rockall scores were compared to four ML models: logistic regression with regularization, RF classifier, GBM, and voting classifier. The RF model predicted death with the highest accuracy, showing a substantial improvement over conventional techniques (AUC: RF 0.92 vs. Glasgow-Blatchford score 0.71) [32]. Sarajlic P et al. developed an ML model for predicting upper gastrointestinal hemorrhage after an acute myocardial infarction. In 149,477 patients, they employed 25 predictor variables, and an RF-based ML model yielded a C-index of 0.73 [33]. Levi R et al. developed ML models to predict rebleeding in patients admitted to the intensive care unit with gastrointestinal hemorrhage. In 14,620 patients, they employed 20 clinical and pathological characteristics. This study developed an ensemble ML model with an AUC of 0.81 on an internal test dataset [34]. The detailed explanations of each study are demonstrated in Table 2. 

### 3.3. Gastric Cancer

In the context of gastric cancer, Leung W et al. demonstrated in 2020 the effectiveness of an XGBoost model in predicting gastric cancer risk in patients following *Helicobacter pylori* eradication. This study encompassed 26 clinical variables in 89,568 individuals and yielded an internal test AUC of 0.97, surpassing the AUC of the conventional logistic regression model (AUC of 0.90) [35]. Arai J et al. developed an ML model for the customized prediction of stomach cancer incidence based on endoscopic and clinical characteristics. This investigation utilized the GBDT model with eight clinical and biological variables in 1099 patients, resulting in an internal test C-index of 0.84 [36]. Zhou C et al. employed 10 clinical factors to compare five ML models for predicting the recurrence of gastric cancer after surgery in 2012 patients. This study found that logistic regression exhibited the highest accuracy of 0.80, followed by the RF algorithm and GBM [37]. Zhou CM et al. utilized 10 clinicopathological characteristics to develop ML models for predicting lymph node metastases in 1169 patients with postoperative poorly differentiated-type intramucosal gastric cancer. In the internal test, XGBoost achieved the highest accuracy rate (0.95) among the seven algorithmic models [38]. Mirniaharikandehei S et al. employed GBM with a random projection algorithm to develop a non-invasive prediction model for stomach cancer metastases before surgery in 2021. Using five clinical characteristics and abdominal computed tomography (CT) images, this model achieved an accuracy of 71.2%, precision of 65.8%, sensitivity of 43.1%, and specificity of 87.1% in 159 patients [39]. In 2020, Zhou C et al. developed ML models to predict peritoneal metastasis of gastric cancer, using GBM, light GBM, and RF, decision tree and logistic regression models. The study included 1080 individuals and assessed 20 clinical and biochemical factors. In the internal test, GBM and light GBM achieved the highest accuracy (0.91) among the five ML models [40]. Bang et al. developed an ML model for predicting curative resection in undifferentiated-type early gastric cancer prior to endoscopic submucosal dissection. This study included eight clinical and biologic factors and involved 2703 patient in the internal test group and 402 patient in the external test group with undifferentiated-type early gastric cancer. The XGBoost-based ML model performed the best, achieving an internal test accuracy of 93.4% and an external test accuracy of 89.8% [25]. Li M et al. employed Raman spectroscopy along with other ML approaches to differentiate serum samples from stomach cancer patients and healthy controls. In this study, which included 213 serum samples (109 patients and 104 healthy volunteers), the RF model achieved the highest accuracy of 92.8% [41]. Liu D et al. developed ML models to predict post-operative prognosis in gastric cancer patients. This study included 12 clinicopathological variables from 17,690 postoperative gastric cancer patients. The Lasso regression-based model achieved an AUC of approximately 0.8 in both the internal and external test sets [42]. Chen Y et al. developed ML models for predicting major pathological responses to neoadjuvant treatment in patients with advanced gastric cancer. In this study of 221 individuals, 15 clinicopathological characteristics were employed. The Lasso regression-based model demonstrated good predictive accuracy with a C-index of 0.763 [43]. Using a large national dataset, Rahman SA et al. built an ML prediction model for overall survival following surgery in gastric cancer using non-linear random survival forests bootstrapping. This study utilized 29 clinical and pathological factors in 2931 patients. At 5 years of survival, the prediction performance revealed a time-dependent AUC of 0.80 and a C-index of 0.76 [44]. Wang S et al. developed an ensemble multi-layer neural network model to classify Borrmann classification in advanced stomach cancer. In the external test, AUC was 0.70 for Borrmann I/II/III versus IV and 0.73 for Borrmann II versus III [45]. Table 3 provides a detailed description of each study.

### 3.4. Gastrointestinal Tumors and Cancers

Christopherson KM et al. developed ML models to predict 30-day unplanned hospitalization in gastrointestinal malignancies. This study included 1341 consecutive patients undergoing gastrointestinal radiation treatment. The GBDT-based model exhibited the best prediction performance with an AUC of 0.82 [46]. Shimizu H et al. created a comprehensive atlas of predictive genes using an integrated meta-analysis of colorectal cancer populations. They employed a machine learning strategy based on a Lasso regression model to develop a universal molecular prognostic score based on the expression state of only 16 genes. This score was tested with independent datasets and proved to be predictive [47]. Wang J et al. developed ML models for distinguishing gastric schwannomas from gastrointestinal stromal tumors using CT images and eight clinical factors. The logistic regression-based ML model achieved the highest AUC of 0.97 in the internal test [48]. Wang M et al. developed ML models for risk classification of gastrointestinal stromal tumors, utilizing CT images in a study involving 324 patients with gastrointestinal stromal tumors. In the external test cohort, the RF-based ML model demonstrated the highest risk stratification accuracy (AUC 0.90) [49]. Table 4 provides a detailed description of each study.

## 4. Challenges and Future Directions for ML Application 

Disease diagnosis is becoming increasingly challenging due to the rising number of possible illnesses resulting from lifestyle changes and modern work environments, which pose significant obstacles to individuals in their daily lives [50]. 

ML has shown significant promise in disease identification and classification by leveraging data. The high prevalence of gastrointestinal disorders, associated mortality rates, and the wealth of data generated through procedures in this field are expected to expand diagnostic and treatment capabilities, fostering innovative therapies [10]. 

Current patient privacy, security, and authentication precautionary measures are deemed insufficient. Overfitting during model training can occur due to two main reasons: excessively large model sizes and inadequate sample data for training. Consequently, the use of synthetic data has gained popularity as a potential approach to enhance research reproducibility and implement differential privacy for protected health information [51]. The goal of data synthesis is to create a dataset that closely resembles the original individual-level data and retrain prediction models [52]. Synthesized data can expedite methodological advancements in medical research and assist in processing high-dimensional and challenging medical data. Given the complexity and high-dimensional nature of patient data, the medical research field has primarily turned to ML techniques [53]. In the medical domain, ML can predict diagnoses, prognoses, and formulate comprehensive treatment plans for newly identified diseases, facilitating the monitoring and assessment of treatment efficacy. ML technology can offer healthcare professionals more accurate and timely solutions [54]. 

Despite achieving high levels of accuracy through sophisticated computations, ML techniques are often characterized by low interpretability, often referred to as their “black-box nature” [25]. Notably, in ML models, there is often a trade-off between accuracy and interpretability [16]. Efforts are currently underway to develop explainable AI analysis, which aims to provide insights into how ML reaches its decisions [25]. The belief is that ML outperforms traditional statistics because it performs complex operations that consider all variables. Further research into explainable AI statistics is expected to shed light on the actual performance of ML models [25]. 

## 5. Large Language Model

The era of conversational AI began in earnest with the introduction of Transformer-based ChatGPT (Chat Generative Pre-trained Transformer, OpenAI Inc., San Francisco, CA, USA) in November 2022 [55] and Bard (Google LLC., Washington, USA) based on a language model called LaMDA, which was announced in March 2023 [56]. AI’s ability to learn data as intended by developers and perform assigned tasks accurately and precisely has fostered trust in its capabilities. However, the challenge of AI generating new data or creating content like writing, music, and paintings posed a different set of hurdles. While AI excelled at predefined tasks, the realm of creativity seemed beyond its grasp. There was little expectation that models not specifically trained for creative tasks could replicate the realm of creativity by learning from large-scale unlabeled data. Nevertheless, the current output of large language models creates the illusion that AI models possess intelligence or cognitive abilities [57].

While we may not have an exact explanation for this phenomenon, it is likely attributed to AI’s enhanced reasoning capabilities as it learns from larger languages and codes. In the future, the performance of these super-sized AI’s foundation models will likely continue to improve. As multimodal AI models trained on extensive data and inaccessible hardware to individual researchers and small companies emerge, there may come a point where simpler models become obsolete, and only specific models remain in use. There are several issues to address before these foundational models can be effectively applied in the medical field, including cost, efficacy validation, privacy and security concerns, and safety concerns like hallucination or the generation of false or harmful information. However, if a robust, feature-rich model is eventually released as open source, it has the potential to be rapidly adopted and widely utilized in the medical field [57].

Large language models are gaining popularity as they possess the ability to generate human-like text [58]. These models have captured the interest of the scientific community due to their potential applications in clinical decision support and academic writing [58]. These chatbot models leverage natural language processing to analyze inquiries and automate responses, simulating human conversation [59]. The next generation of chatbots could assist with medical paperwork tasks and provide answers to crucial questions that may aid in differential diagnosis [59]. However, determining the appropriateness of the responses provided remains a challenge. Large language models are expected to become indispensable tools in real-world clinical settings, especially for tasks like differential diagnosis and research. While they have the potential to enhance our work, it is essential to handle them with care to avoid potential harm [59]. Given that medical data typically comprise multimodal information, including images, language, and test results, it is anticipated that large language models capable of performing various ML analyses will play an increasingly vital role in the future.

## 6. Conclusions

ML models have the potential to significantly improve the quality of interpretation for gastrointestinal disorders. However, it remains to be seen whether these models can be effectively applied to different populations and therapeutic settings. To mitigate inherent biases, rigorous randomized trials and extensive external validation of algorithms across diverse populations are necessary. The landscape of diagnosis and treatment in gastroenterology is poised for dramatic transformation in the coming years, driven by the ongoing advancements in ML. Therefore, it is crucial to comprehend the current state and future potential of this technology for its seamless integration into future medical practice. While the adoption of ML in the field of gastroenterology has been limited, its usage is expected to increase in the future due to its superior and more accurate predictive capabilities compared to traditional statistical methods.

## Figures and Tables

**Table 1:** Summary of clinical studies using machine learning in general subject of gastroenterology.

Reference	Published Year	Aim of Study	Design of Study	Number of Subjects	Type of Machine Learning Model	Input Variables	Outcomes
Klang E et al. [26]	2021	Prediction of outcomes in acute diverticulitis	Retrospective	4497 patients	XGboost	31 clinical and biologic variables	Internal test performance: Sensitivity: 88%, Negative predictive value: 99% External test AUC: 0.85
Yoshii S et al. [27]	2020	Diagnosis of *Helicobacter pylori* infection status based on the Kyoto classification of gastritis	Prospective	498 patients	generalized linear model	16 endoscopic features	Internal test performance: overall diagnostic accuracy: 82.9%
Konishi T et al. [28]	2021	Prediction of postoperative mortality in gastroduodenal ulcer perforation	Retrospective	25,886 patients	Lasso and XGBoost	45 clinical candidate predictors	Internal test performance: Lasso (AUC: 0.84) XGBoost (AUC: 0.88)
Liu Y et al. [29]	2021	Prediction of patient risk of upper gastrointestinal lesions to identify high risk for endoscopy	Retrospective	620 patients	Support vector machine	48 clinical symptoms, serological results, and pathological variables	Internal test accuracy: 91.2%

XGBoost, extreme gradient boosting; AUC, area under the curve.

**Table 2:** Summary of clinical studies using machine learning in gastrointestinal hemorrhage.

Reference	Published Year	Aim of Study	Design of Study	Number of Subjects	Type of Machine Learning Model	Input Variables	Outcomes
Shung DL et al. [30]	2019	Risk of hospital-based intervention or death in patients with upper gastrointestinal hemorrhage	Retrospective	2357 patients	XGBoost	24 clinical and biologic variables	Internal test AUC: 0.91 External test AUC: 0.90
Herrin J et al. [31]	2021	Prediction of gastrointestinal hemorrhage in patients receiving antithrombotic treatment	Retrospective	306,463 patients	Regularized Cox proportional hazards regression model and the XGBoost model	32 clinical variables	Internal test AUC: 0.67 at 6 months, 0.66 at 12 months
Seo DW et al. [32]	2020	Prediction of adverse events in stable non-variceal gastrointestinal hemorrhage	Retrospective	1439 patients	Random forest	38 clinicopathological variables	Internal test AUC: 0.92
Sarajlic P et al. [33]	2021	Predictors of upper gastrointestinal hemorrhage following acute myocardial infarction	Nationwide cohort Prospective	149,477 (acute myocardial infarction patients with antithrombotic therapy)	Random forest	25 predictor variables	C-index: 0.73
Levi R et al. [34]	2020	Prediction of rebleeding in patients admitted to the intensive care unit with gastrointestinal hemorrhage	Retrospective	14,620 patients	Ensemble machine learning model	20 clinical and pathological variables	Internal test AUC: 0.81

XGBoost, extreme gradient boosting; AUC, area under the curve.

**Table 3:** Summary of clinical studies using machine learning in gastric cancer.

Reference	Published Year	Aim of Study	Design of Study	Number of Subjects	Type of Machine Learning Model	Input Variables	Outcomes
Leung W et al. [35]	2020	Prediction of gastric cancer risk in patients after *Helicobacter pylori* eradication	Retrospective	89,568 patients	XGBoost	26 clinical variables	Internal test performance: AUC: 0.97 Sensitivity: 98.1% Specificity: 93.6%
Arai J et al. [36]	2021	Prediction of gastric cancer incidence	Retrospective	1099 patients	GBDT	8 clinical and biological variables	C-index: 0.84
Zhou C et al. [37]	2021	Prediction for recurrence of gastric cancer after operation	Retrospective	2012 patients	Logistic regression	10 clinical variables	Internal test accuracy: 0.801
Zhou CM et al. [38]	2021	Prediction of lymph node metastasis of poorly differentiated-type intramucosal gastric cancer	Retrospective	1169 patients with postoperative gastric cancer	XGBoost	10 clinicopathological variables	Internal test accuracy: 0.95
Mirniaharikandehei S et al. [39]	2021	Prediction of gastric cancer metastasis before surgery	Retrospective	159 patients	GBM with random projection algorithm	5 clinical variables and abdominal computed tomography images	Internal test performance: Accuracy: 71.2% Precision: 65.8% Sensitivity: 43.1% Specificity: 87.1%
Zhou C et al. [40]	2020	Prediction of peritoneal metastasis of gastric cancer	Retrospective	1080 patients	GBM, light GBM	20 clinical and biological variables	Internal test accuracy: 0.91
Bang et al. [25]	2021	Prediction the possibility of curative resection in undifferentiated-type early gastric cancer prior to endoscopic submucosal dissection	Retrospective	3105 undifferentiated-type early gastric cancers	XGboost	8 clinical variables	External test accuracy: 89.8%
Li M et al. [41]	2021	Differentiate serum samples from stomach cancer patients and healthy controls	Retrospective	109 patients (including 35 in stage I, 14 in stage II, 35 in stage III, and 25 in stage IV) 104 health volunteers	Random forest in conjunction with Raman spectroscopy	Serum samples	Internal test performance: Accuracy: 92.8% Sensitivity: 94.7% Specificity: 90.8%
Liu D et al. [42]	2021	Prediction of prognosis of postoperative gastric cancer	Retrospective	17,690 patients with gastric cancer (additional external test set: 955)	Lasso regression	12 clinicopathological variables	Internal test and external test AUC: 0.8
Chen Y et al. [43]	2021	Prediction of major pathological response to neoadjuvant chemotherapy in advanced gastric cancer	Retrospective	221 patients	Lasso regression	15 clinicopathological variables	C-index: 0.763
Rahman SA et al. [44]	2021	Prediction of long-term survival after gastrectomy	Retrospective	2931 patients	Non-linear random survival forests bootstrapping	29 clinical and pathological variables	Internal test performance: time-dependent AUC at 5 years: 0.80 C-index: 0.76
Wang S et al. [45]	2020	Borrmann classification in advanced gastric cancer	Retrospective	597 AGC patients (additional 292 patients for external test)	Ensemble multi-layer neural network	Computed tomography images (Borrmann I/II/III vs. IV and Borrmann II vs. III)	External test performance: Borrmann I/II/III vs. IV AUC: 0.7, Borrmann II vs. III AUC: 0.73

XGBoost, extreme gradient boosting; AUC, area under the curve; GBDT, gradient-boosting decision tree; GBM, gradient-boosting machines.

**Table 4:** Summary of clinical studies using machine learning in gastrointestinal tumors and cancers.

Reference	Published Year	Aim of Study	Design of Study	Number of Subjects	Type of Machine Learning Model	Input Variables	Outcomes
Christopherson KM et al. [46]	2020	Prediction of 30-day unplanned hospitalization for gastrointestinal malignancies	Prospective	1341 patients (consecutive patients undergoing gastrointestinal radiation treatment)	GBDT	787 predefined candidate clinical and treatment variables	Internal test performance: AUC: 0.82
Shimizu H et al. [47]	2021	Development of universal molecular prognostic score based on the expression state of 16 genes in colorectal cancer	Retrospective	Over 1200 patients	Lasso regression	Gene scoring	Application of established genetic universal prognostic classifier for patients with gastric cancers and showed acceptable prediction in Kaplan–Meyer curve
Wang J et al. [48]	2020	Differentiation of gastric schwannomas from gastrointestinal stromal tumors using computed tomography images	Retrospective	188 patients/ 49 patients with schwannomas and 139 patients with gastrointestinal stromal tumors	Logistic regression	8 clinical characteristics and computed tomography findings	Internal test performance: AUC: 0.97
Wang M et al. [49]	2021	Prediction of risk stratification for gastrointestinal stromal tumors	Retrospective	180 patients with gastrointestinal stromal tumors (additional 144 patients for external test)	Random forest	Computed tomography images (Top 10 features with importance value above 5)	External test performance: AUC: 0.90

AUC, area under the curve; GBDT, gradient-boosting decision tree.

## Data Availability

Not applicable.
